# Prognostic value of CD20 antigen mediated immune checkpoint inhibition in patients with acute or chronic lymphocytic leukemia: A protocol for systematic review

**DOI:** 10.1097/MD.0000000000028868

**Published:** 2022-02-18

**Authors:** Zekhethelo A. Mkhwanazi, Snenhlanhla A. Mfusi, Bongani B. Nkambule

**Affiliations:** School of Laboratory Medicine and Medical Sciences (SLMMS), College of Health Sciences, University of KwaZulu-Natal, Durban, South Africa.

**Keywords:** acute lymphoblastic leukemia, chemoimmunotherapy, chronic lymphocytic leukemia, rituximab

## Abstract

**Background:**

The addition of rituximab to standard chemotherapy has been shown to improve response rates in patients with acute or chronic lymphocytic leukemia. However, the prognostic factors associated with progression-free survival in rituximab treated patients with lymphocytic leukemias remains unclear. We will perform a comprehensive systematic review and meta-analysis on available data on prognostic factors associated with the clinical outcomes of patients with acute and chronic lymphocytic leukemia.

**Methods and analysis:**

This protocol for a systematic review and meta-analysis of prognostic factors has been prepared following the Preferred Reporting Items for Systematic Review and Meta-Analysis Protocols (PRISMA-P) 2015 guidelines. Electronic databases will be searched using keywords related to the objectives of this review. This systematic review and meta-analysis will include published randomized clinical trials, observational, prospective, and retrospective comparative cohorts. Two reviewers (ZAM and SAM) will independently screen studies, with a third reviewer consulted in cases of disagreements using a defined inclusion and exclusion criteria. Data items will be extracted using a predefined data extraction sheet. Moreover, the risk of bias and the quality of evidence were independently assessed using the quality in prognostic studies tool (QUIPS). The I2 and chi squared statistical tests will be used to analyze statistical heterogeneity across studies. An I2 values of > 50% will be considered substantial. All data analysis will be performed using STATA 16.0 (StataCorp LP, TX, USA). The outcomes examined will be progression-free and overall survival.

**Ethics and dissemination:**

No ethical approval will be required and the findings of this meta-analysis will be published in a peer-reviewed journal.

**Systematic review registration:**

International prospective Register of Systematic Reviews (PROSERO) number: CRD42021218997.

**Key Points**

- This systematic review and meta-analysis will be the first to synthesise the prognostic factors associated with Rituximab and the effectiveness of rituximab-based therapy in patients with acute and chronic lymphocytic leukemia.
- To our knowledge, this systematic review will offer a robust assessment of the evidence and quality of clinical and cell-based traditional and novel prognostic factors.
- The various disease stage and the severity of the disease in CLL/ALL patients included in the studies will be one of the limitations of this systematic review and meta-analysis.

## 1 Introduction

Chronic lymphocytic leukemia (CLL) is characterized by progressive proliferation and accumulation of functionally incompetent lymphocytes in the peripheral blood, bone marrow, lymph nodes, and spleen.^[^1^,^^2^^]^ CLL is predominantly due to profound defects in B lymphocytes and is also characterized by T-cell exhaustion,^[3]^ whereas acute lymphocytic leukemia (ALL) involves aggressive accumulation of blasts in the bone marrow and has been the primary cause of cancer-related mortalities in children and adolescents.^[^2^,^^4^^]^ CLL is considerably more severe in adult patients over the age of 65 years^[5]^ and men are disproportionally affected, with a higher incidence than women.^[^6^–^9^]^ This can be attributed to gender-specific hormonal differences or the variance in IGVH gene usage and mutational status.^[10]^

Several clinical and genetic-based prognostic markers have been established. In fact, the CLL international prognostic index (CLL-IPI) includes validated clinical, genetic, and laboratory features in the prognostication of patients with CLL.^[11]^ The incidence of CLL varies widely across geographic locations, with a high distribution of chronic lymphocytic leukemia in most European and North American countries.^[9]^ In comparison, ALL cases remain relatively high in North America, Northern and Western Europe, while lower rates are apparent in Asian and African populations^[12]^ Notably, there is minimal data on the incidence or prevalence of lymphocytic leukemia in African countries. Based on currently available data on the prevalence of lymphocytic leukemia in multi-ethnic studies, CLL seems to affect Europeans more than Africans. The discrepancy in patient outcomes across racial and ethnic groups have been presented in some studies.^[^12^–^19^]^ In studies including ethnic minorities, a poor overall survival rate was reported when compared with their white counterparts. Disparities could be partly attributed to demographic variables, varied biological responses, environmental and genetic factors as well as socioeconomic status between these racial groups.^[^20^,^^21^^]^ Patient outcomes have improved remarkably over the years due to the adoption of risk-based therapy that is determined by patient characteristics and leukemia phenotype at diagnosis.^[17]^ For several decades, high-dose standard chemotherapy has been the primary treatment, however, this regimen has no demonstrable improvement in overall survival.^[22]^

Recently, a novel approach using monoclonal antibodies aimed at restoring the immune system by targeting immune checkpoints proteins has provided effective and complementary treatments.^[23]^ The use of fludarabine, cyclophosphamide, and rituximab (FCR) therapy significantly improves the progression-free and overall survival of patients with CLL.^[24]^ To date, there are currently no published systematic review and meta-analysis providing cumulative evidence on the predictors of mortality or poor patient outcomes of patients with CLL on rituximab. This systematic review and meta-analysis will assess the available studies reporting on the prognostic factors and efficacy of combined chemoimmunotherapy containing rituximab. We will further synthesize the novel prognostic factors associated with poor clinical outcomes.

### 1.1 Research question

Does combining rituximab with standard chemotherapy improve progression-free survival of patients with CLL?

Which clinical and laboratory features are associated with poor prognosis and variable patient outcomes following rituximab therapy?

### 1.2 Objectives

To assess progression-free and overall survival in patients with CLL on rituximab or immunochemotherapy containing rituximab. Furthermore, to determine novel prognostic factors (cell-based proteins: CD38, ZAP70, CD49D; Serology: β2 M, thymidine kinase, lactate dehydrogenase, Interleukin 8) associated with poor patient outcomes.

## 2 Methods

This protocol for a systematic review and meta-analysis has been prepared in accordance with Preferred Reporting Items for Systematic Review and Meta-Analysis Protocols 2015 (PRISMA-P) guidelines. The protocol was registered with the online PROSPERO registry (CRD42021218997).

### 2.1 Study design

In this review, we will include randomized control trials, prospective and retrospective comparative cohorts.

#### 2.1.1 Inclusion criteria

Only primary studies assessing the prognosis (progression-free and overall survival) of patients with CLL on rituximab-based therapy will be included. The search will be restricted to full-text human studies written in English.

#### 2.1.2 Exclusion criteria

Cross-sectional and case-control studies will be excluded. In addition, review articles, letters, and editorials will be excluded.

### 2.2 Population

Patients with CLL on rituximab-based therapy, will be included.

### 2.3 Index prognostic factor

We will consider the predictive factors included in the widely used CLL International Prognostic Index (CLL-IPI).^[25]^ We will also consider the predictive factors used in the German CLL Study Group (GCLLSG),^[26]^ the MD Anderson Cancer Center (MDACC) nomogram^[27]^ predictive models.

### 2.4 Comparators

The comparators will include patients receiving standard therapy or usual care.

### 2.5 Outcomes

The primary outcome will be the overall 5-year overall survival, and the secondary outcome will include 2- to 4-year progression-free survival.

### 2.6 Timing and setting

The predictive information and measurements at diagnosis and initiation of treatment will be considered. Moreover, we will include studies reporting on inpatient and outpatient cohorts.

#### 2.6.1 Search strategy and study selection

The search strategy will be developed using medical subject headings (MeSH) for MEDLINE, and this will be adapted to EBSCOhost search headings terms. We will search the databases from inception to February 28, 2021. The search strategy will consist of search terms that include chronic lymphocytic leukemia, acute lymphoblastic leukemia, rituximab (Supplementary file 1).

### 2.7 Data management

#### 2.7.1 Data collection process

The reviewers (ZAM and SAM) will develop a structured data extraction form that will be used in the data extraction process. Mendeley referencing manager will be used in this systematic review. The screening of the articles will be independently assessed by 2 reviewers (ZAM and SAM).

#### 2.7.2 Data items

The 2 reviewers (ZAM and SAM) will extract data items using the data items defined in the checklist for critical appraisal and data extraction for systematic reviews of prediction modelling studies for prognostic factors (CHARMS-PF).^[28]^ This will include the following information: source of data, participant description, the study dates, sample size, predicted outcome including outcome measures (hazards, odds ratio), candidate predictors, handling of missing data, modeling method, and model performance.

#### 2.7.3 Data simplification

Studies will be grouped according to the type of lymphocytic leukemia (CLL or ALL). In addition, studies will be grouped based on the, gender ratio, age of participants and chemotherapy regimen used (e.g., fludarabine, cyclophosphamide, ibrutinib), duration of intervention and follow-up.

#### 2.7.4 Risk of bias in individual studies

To assess the potential risk of bias in the included studies the quality in prognostic studies (QUIPS) tool will be used.^[29]^ Two authors (ZAM and SAM) will independently assess the included studies based on the 6 domains of the tool. In a case of disagreements, a third reviewer (BBN) will be consulted for arbitration.

### 2.8 Data synthesis

A summary of findings table (SoF) will be used to provide a synthesis of the main outcomes of included studies. Furthermore, if the included studies are homogeneous in terms of the type of lymphocytic leukemia treated, therapy used, and participant characteristics, data will be analyzed using a fixed-effects model. All data analysis will be performed using R statistical software (The R foundation for statistical computing, Vienna, Austria). The *I* ^2^ and chi-squared statistical tests will be used to analyze statistical heterogeneity between studies.^[^30^,^^31^^]^ An *I* ^2^ value of >50% will be considered substantial heterogeneity.^[32]^

### 2.9 Subgroup analysis

To explore the sources of heterogeneity within the included studies, we will perform a subgroup analysis based on the study-level characteristics, including the risk of bias of the included studies, geographic location, intervention type (rituximab plus chemotherapy [R-Chemo] and chemotherapy regimens). Lastly, the reported measure of progression-free and overall survival will also be considered in the subgroup analysis.

### 2.10 Confirmation of predictive factors

The prognostic factors will be confirmed based on the consistency of the overall direction of the effect across the included studies. In addition, adjusted effect sizes that remain statistically significant (*P* < .05) after adjusting for covariates and multivariate analysis will be considered as confirmed.

#### 2.10.1 Quality assessment of the cumulative evidence

The quality and strength of the evidence of the confirmed prognostic factors will be evaluated by 2 independent reviewers (ZAM, SAM) using the Grading of Recommendations Assessment Development and Evaluation approach (GRADE).^[33]^

### 2.11 Patient and public involvement

There is no patient or public involvement in the process of conducting this study.

## 3 Discussion

Rituximab-based therapy has demonstrated therapeutic benefits in the treatment of patients with lymphocytic leukemia. This systematic review and meta-analysis of prognostic factors will provide a comprehensive synthesis of studies reporting on progression-free survival in patients with CLL on a rituximab-based regimen compared with standard chemotherapy. Moreover, this review will provide a synthesis of traditional and novel cell-based prognostic factors. To our knowledge, this will be the first meta-analysis to evaluate the efficacy of R-chemo and predictive factors associated with progression-free survival in patients with ALL and CLL compared with chemotherapy alone. Findings from this study will provide insight into the prognostic factors in patients with CLL following R-chemo and will assist in the patient management and prognostication.

## Author contributions

Zekhethelo A. Mkhwanazi, Snenhlanhla A. Mfusi, Bongani B. Nkambule conceptualized, designed the study, and drafted the protocol. Zekhethelo A. Mkhwanazi and Bongani B. Nkambule wrote the first draft. All authors approved the final manuscript. Bongani B. Nkambule provided supervision and is the guarantor of the review.

**Conceptualization:** Zekhethelo A. Mkhwanazi, Bongani B. Nkambule.

**Data curation:** Zekhethelo A. Mkhwanazi.

**Methodology:** Bongani B. Nkambule.

**Supervision:** Bongani B. Nkambule.

**Writing – original draft:** Zekhethelo A. Mkhwanazi.

**Writing – review & editing:** Snenhlanhla A. Mfusi, Bongani B. Nkambule.

## Supplementary Material

Supplemental Digital Content

## References

[1] Rodrigues CA Gonçalves MV Ikoma MRV. Diagnosis and treatment of chronic lymphocytic leukemia: recommendations from the Brazilian Group of Chronic Lymphocytic Leukemia. Rev Bras Hematol Hemoter 2016;38:346–57. 27863764 10.1016/j.bjhh.2016.07.004 PMC5119662

[2] Xiao X Ye X Xu C Huang J. Successful alternative treatment for relapsed adult acute lymphoblastic leukemia with dendritic cells-cytokine-induced killer cells combined with a rituximab-based regimen. Onco Targets Ther 2018;11:7555–8. 30464503 10.2147/OTT.S177503 PMC6214580

[3] Riches JC Davies JK Mcclanahan F. T cells from CLL patients exhibit features of T-cell exhaustion but retain capacity for cytokine production. Blood 2013;121:1612–21. 23247726 10.1182/blood-2012-09-457531 PMC3587324

[4] Heatley SL Sadras T Kok CH. High prevalence of relapse in children with Philadelphia-like acute lymphoblastic leukemia despite risk-adapted treatment. Haematologica 2017;102:e490–3. 28935844 10.3324/haematol.2016.162925 PMC5709119

[5] Gribben JG. Chronic lymphocytic leukemia: planning for an aging population. Expert Rev Anticancer Ther 2010;10:1389–94. 20836674 10.1586/era.10.127 PMC4241366

[6] Siegel R Naishadham D Jemal A. Cancer statistics, 2013. CA Cancer J Clin 2013;63:11–30. 23335087 10.3322/caac.21166

[7] Catovsky D Fooks J Richards S. Prognostic factors in chronic lymphocytic leukaemia: the importance of age, sex and response to treatment in survival A report from the MRC CLL 1 trial. M.R.C. Working party on Leukemia in adults. Br J Haematol 1989;72:141–9. 2757960 10.1111/j.1365-2141.1989.tb07674.x

[8] Mandelli F de Rossi G Mancini P. Prognosis in chronic lymphocytic leukemia: a retrospective multicentric study from the GIMEMA group. J Clin Oncol 1987;5:398–406. 3819805 10.1200/JCO.1987.5.3.398

[9] Miranda-Filho A Piñeros M Ferlay J Soerjomataram I Monnereau A Bray F. Epidemiological patterns of leukaemia in 184 countries: a population-based study. Lancet Haematol 2018;5:e14–24. 29304322 10.1016/S2352-3026(17)30232-6

[10] Catovsky D Wade R Else M. The clinical significance of patients’ sex in chronic lymphocytic leukemia. Haematologica 2014;99:1088–94. 24658818 10.3324/haematol.2013.101378 PMC4040913

[11] Bergmann MA Eichhorst BF Busch R. Prospective evaluation of prognostic parameters in early stage chronic lymphocytic leukemia (CLL): results of the CLL1-protocol of the German CLL Study Group (GCLLSG). Am Soc Hematol 2007;110:625.

[12] Parkin DM Muir CS Whelan SL. Cancer Incidence in Five Continents, Vol. 6. 1992;Lyon: IARC Scientific Publications, 120. 1284606

[13] Shenoy PJ Malik N Sinha R. Racial differences in the presentation and outcomes of chronic lymphocytic leukemia and variants in the United States. Clin Lymphoma Myeloma Leuk 2011;11:498–506. 21889433 10.1016/j.clml.2011.07.002

[14] Pui C-H Boyett JM Hancock ML. Outcome of treatment for childhood cancer in Black as compared with white children. The St Jude children's research hospital 1962 through 1992. JAMA 1995;273:633–7. 7844873

[15] Bhatia S Sather HN Heerema NA Trigg ME Gaynon PS Robison LL. Racial and ethnic differences in survival of children with acute lymphoblastic leukemia. Blood 2002;100:1957–64. 12200352 10.1182/blood-2002-02-0395

[16] Pollock BH DeBaun MR Camitta BM. Racial differences in the survival of childhood B-precursor acute lymphoblastic leukemia: a paediatric oncology group study. J Clin Oncol 2000;18:813–23. 10673523 10.1200/JCO.2000.18.4.813

[17] Kadan-Lottick NS Ness KK Bhatia S Gurney JG. Survival variability by race and ethnicity in childhood acute lymphoblastic leukemia. JAMA 2003;290:2008–14. 14559954 10.1001/jama.290.15.2008

[18] Liu L Krailo M Reaman GH Bernstein L. Childhood cancer patients’ access to cooperative group cancer programs: a population-based study. Cancer 2003;97:1339–45. 12599243 10.1002/cncr.11192

[19] Goggins WB Lo FFK. Racial and ethnic disparities in survival of US children with acute lymphoblastic leukemia: evidence from the SEER database 1988-2008. Cancer Causes Control 2012;23:737–43. 22450738 10.1007/s10552-012-9943-8

[20] Schillinger JA Grosclaude PC Honjo S Quinn MJ Sloggett A Coleman MP. Survival after acute lymphocytic leukaemia: effects of socioeconomic status and geographic region. Arch Dis Child 1999;80:311–7. 10086933 10.1136/adc.80.4.311 PMC1717889

[21] Ruchlemer R Polliack A. Geography, ethnicity and “roots” in chronic lymphocytic leukemia. Leuk Lymphoma 2013;54:1142–50. 23121522 10.3109/10428194.2012.740670

[22] Jaglowski S Jones JA. Choosing first-line therapy for chronic lymphocytic leukemia. Expert Rev Anticancer Ther 2011;11:1379–90. 21929312 10.1586/era.11.132 PMC4341916

[23] Ma K Jin Q Wang M Li X Zhang Y. Research progress and clinical application of predictive biomarker for immune checkpoint inhibitors. Expert Rev Mol Diagn 2019;19:517–29. 31079502 10.1080/14737159.2019.1617702

[24] Keating MJ O’Brien S Albitar M. Early results of a chemoimmunotherapy regimen of fludarabine, cyclophosphamide, and rituximab as initial therapy for chronic lymphocytic leukaemia. J Clin Oncol 2005;23:4079–88. 15767648 10.1200/JCO.2005.12.051

[25] An international prognostic index for patients with chronic lymphocytic leukaemia (CLL-IPI): a meta-analysis of individual patient data. Lancet Oncol 2016;17:779–90. 27185642 10.1016/S1470-2045(16)30029-8

[26] Tam CS Seymour JF. A new prognostic score for CLL. Blood 2014;124:01–2. 10.1182/blood-2014-05-575407 24993873

[27] Molica S Mauro FR Callea V. The utility of a prognostic index for predicting time to first treatment in early chronic lymphocytic leukemia: the GIMEMA experience. Haematologica 2010;95:464–9. 19903673 10.3324/haematol.2009.011767 PMC2833077

[28] Moons KGM de Groot JAH Bouwmeester W. Critical appraisal and data extraction for systematic reviews of prediction modelling studies: the CHARMS Checklist. PLoS Med 2014;11:e1001744. 25314315 10.1371/journal.pmed.1001744 PMC4196729

[29] Hayden JA Co P. Evaluation of the quality of prognosis studies in systematic reviews. Ann Internal Med Acad 2006;144:427–38. 10.7326/0003-4819-144-6-200603210-00010 16549855

[30] Higgins JPT Altman DG Gøtzsche PC. The Cochrane Collaboration's tool for assessing risk of bias in randomised trials. BMJ 2011;343:d5928. 22008217 10.1136/bmj.d5928 PMC3196245

[31] Cochran WG. The combination of estimates from different experiments. Biometrics 1954;10:101–29.

[32] Schroll JB Moustgaard R Gøtzsche PC. Dealing with substantial heterogeneity in Cochrane reviews. Cross-sectional study. BMC Med Res Methodol 2011;11:22. 21349195 10.1186/1471-2288-11-22 PMC3056846

[33] Balshem H Helfand M Schünemann HJ. GRADE guidelines: 3. Rating the quality of evidence. J Clin Epidemiol 2011;64:401–6. 21208779 10.1016/j.jclinepi.2010.07.015

