# Supplementary material for: Prognostic value of CD20 antigen mediated immune checkpoint inhibition in patients with acute or chronic lymphocytic leukemia: A protocol for systematic review
Source: Medicine (Baltimore). 2022 Feb 18;101(7):e28868. doi: 10.1097/MD.0000000000028868 (PMC9282114; doi:10.1097/MD.0000000000028868)
Supplement: Supplemental Digital Content [file medi-101-e28868-s001.doc]

| **Search terms** | **Synonyms/associated terms** | **Hits** |
| --- | --- | --- |
| **Concept 1**  "Chronic lymphocytic leukaemia"[MeSH] | Chronic lymphocytic leukaemia [text]; CLL | 23 862 |
| **Concept2**  “Prognosis” [MeSH] | Prognostication [Text];  Risk stratification [Text]; Outcomes [text] | 794,891 |
| **Concept 3**  “Rituximab” [MeSH] | Rituxan, Anti-CD20, immunotherapy | 25,346 |

Table 1: Supplementary Search strategy run as ran on Medline database via EBSCOhost, 31 March 2021.

Combine concept 1,2 and 3
